# Supplementary material for: Homologous Recombination-Enhancing Factors Identified by Comparative Transcriptomic Analyses of Pluripotent Stem Cell of Human and Common Marmoset
Source: Cells. 2022 Jan 21;11(3):360. doi: 10.3390/cells11030360 (PMC8834151; doi:10.3390/cells11030360)
Supplement: Supplementary file 1 [file cells-11-00360-s001.zip › Supplementary_Material.pdf]

Supplementary Material

Supplementary Figures and Legends

Figure S1

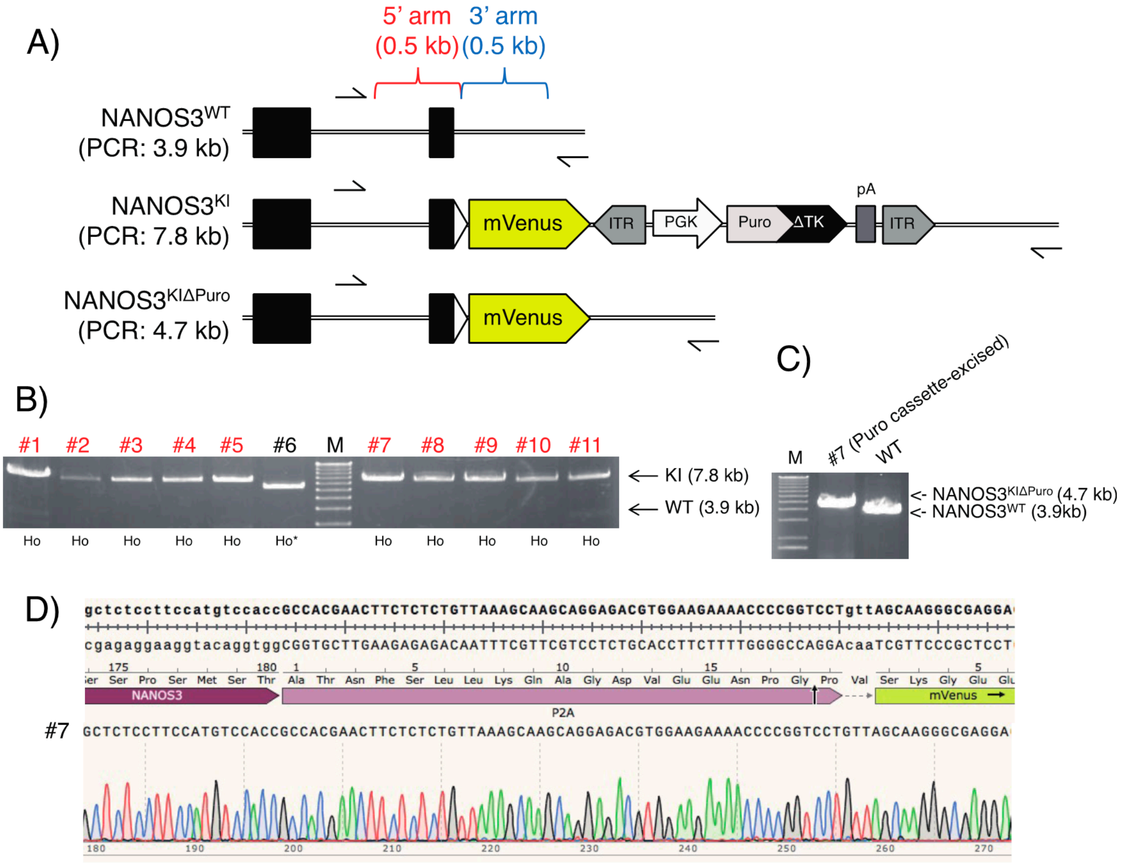

Figure S1. Gene targeting in the marmoset *NANOS3* gene locus. (**A**) Graphical schematics of the wild-type (WT) marmoset *NANOS3* allele (NANOS3^{WT}; top), knock-in *NANOS3* one (NANOS3^{KI}; middle) and puromycin cassette-excised one (NANOS3$^{KI\Delta Puro}$; bottom). Black boxes and arrows indicate endogenous exons of the *NANOS3* gene and primer binding sites (externally from the homology regions) for genotyping PCR. White triangles indicate self-cleaving porcine teschovirus-1 2A sequence (P2A). ITR, excisable *Piggybac transposase* inverted terminal sequence; Puro ΔTK, puromycin resistance gene fused to the N-terminus truncated *thymidine kinase-1*. (**B**) Genotyping PCR analysis of puromycin-resistant cmES cell clones following transfection of the *NANOS3-Venus* targeting vector and Cas9/gRNA vector. Ho, homozygous KI. All analyzed (eleven) clones harbored homozygous NANOS3^{KI} alleles, although the clone #6 (Ho*) harbored aberrant (truncated) recombinant alleles homozygously. 1 Kb Plus DNA Ladder (Thermo Fisher Scientific) was used as a DNA marker (shown as “M”). (**C**) Genotyping PCR analysis of a puromycin cassette-excised knock-in cmES cell clone (originally from the #7 clone

in Figure S1B) with a WT control. Detailed procedures of cassette excision were described previously [1]. (**D**) DNA sequencing analysis of the recombinant alleles of the homozygous knock-in clone #7 (shown in Figure S1B). We confirmed *P2A-Venus* was precisely integrated to the direct downstream of the endogenous *NANOS3* coding sequence.

Figure S2

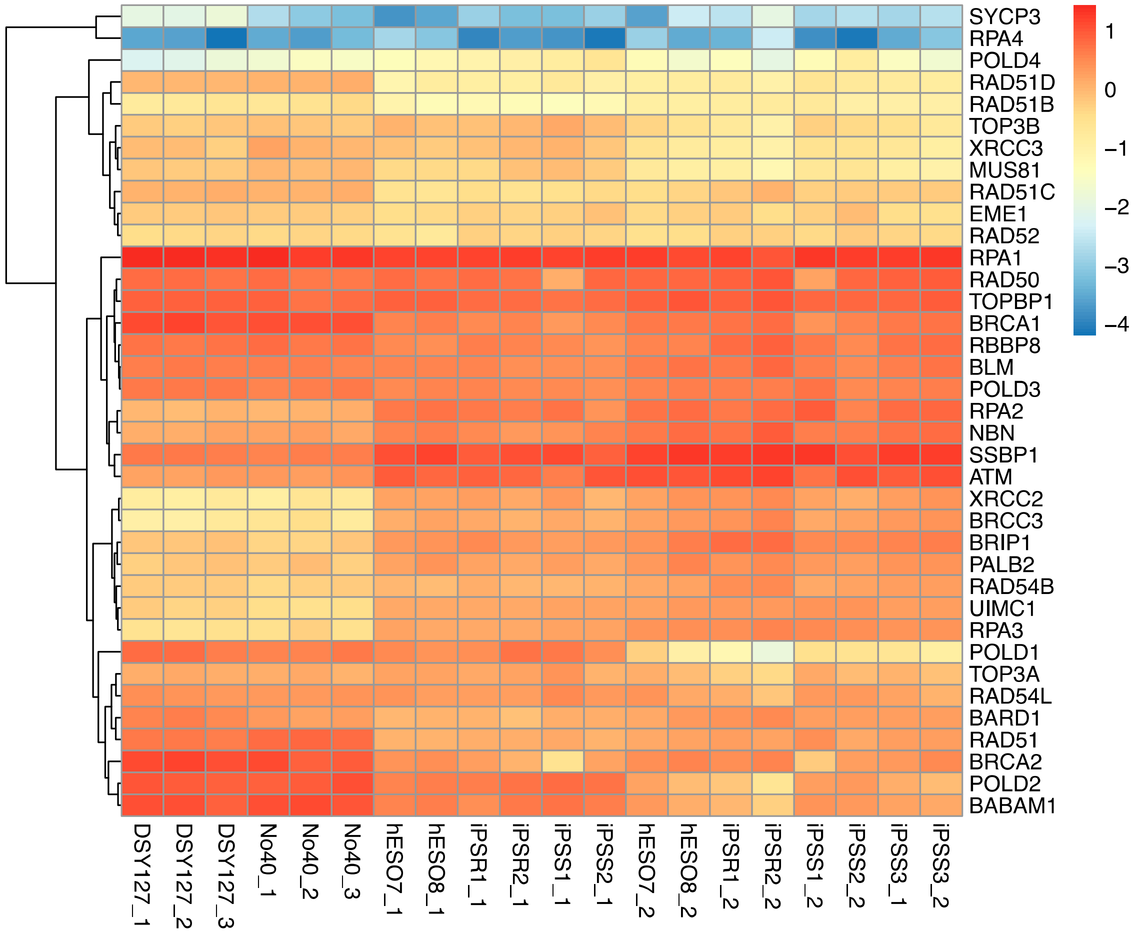

Figure S2. Normalized genes expression of HR-related genes in human and marmoset PSCs. The expression levels were treated with log10.

Figure S3

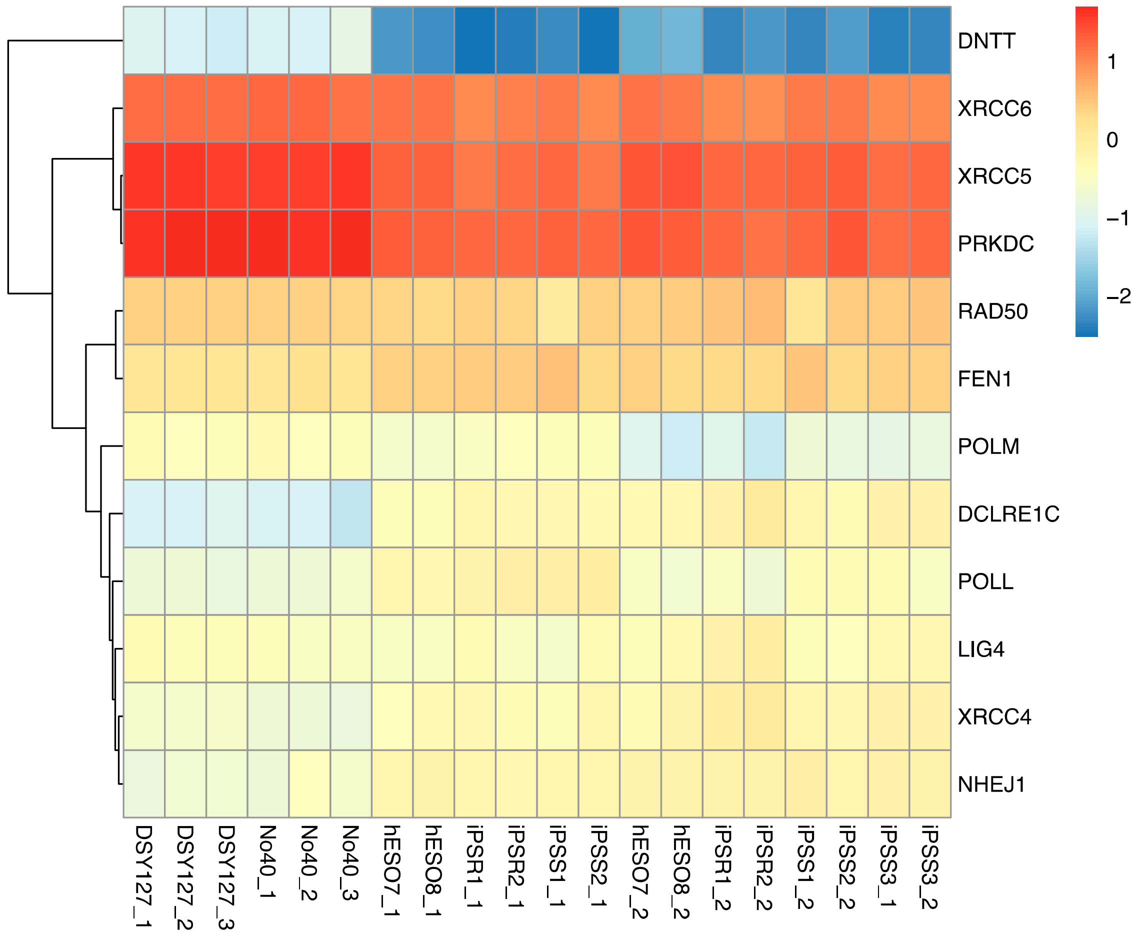

Figure S3. Normalized genes expression of NHEJ-related genes in human and marmoset PSCs. The expression levels were treated with log10.

Figure S4

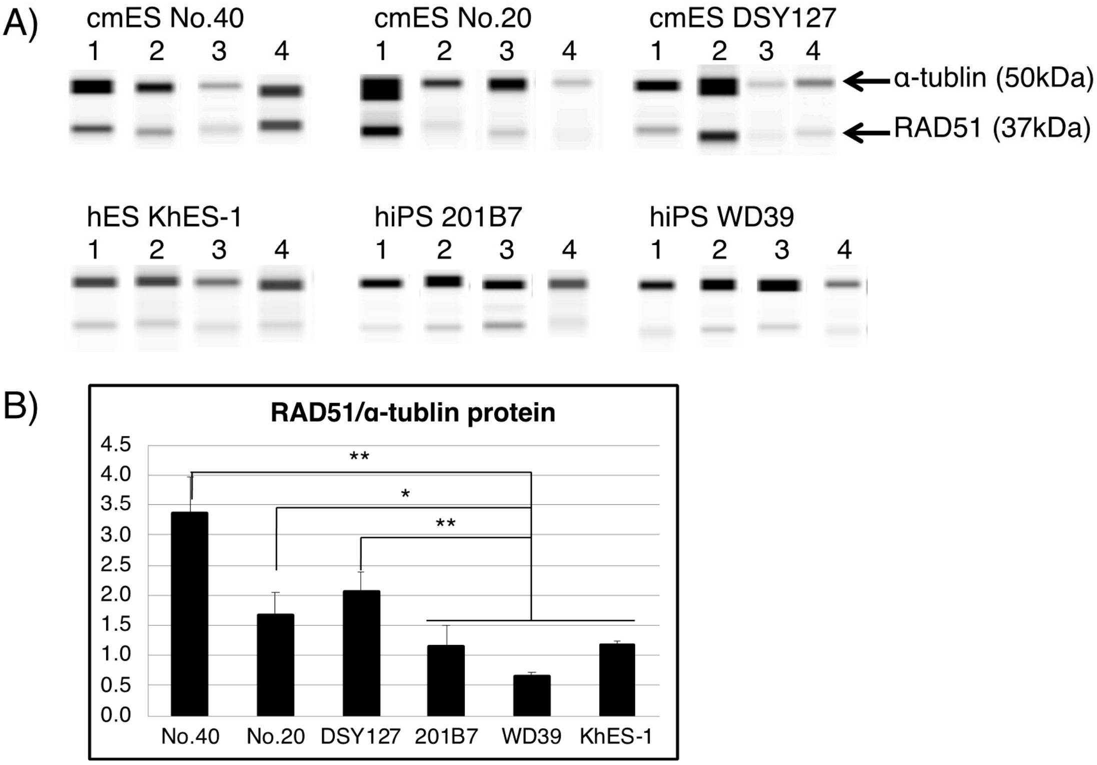

Figure S4. Western blotting analysis of RAD51 protein in human and marmoset PSCs. (**A**) Raw data of digital electrophoresis simultaneously detecting RAD51 (37 kDa) and α-tubulin (50 kDa) proteins. (**B**) Average RAD51 expression (normalized by α-tubulin) in respective PSC lines.

Figure S5

A)

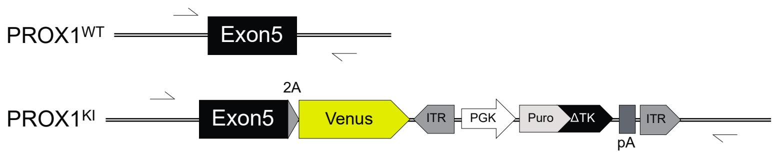

B)

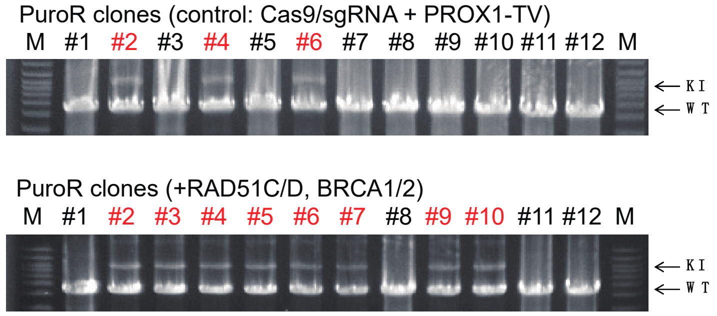

C)

Condition (PROX1-TV + Cas9/sgRNA)	Genotype of PuroR clones (KI/total)
Control	3 /12
+RAD51	5 /12
+RAD51C	6 /12
+RAD51D	7 /12
+BRCA1	8 / 12
+BRCA2	7 /12
+RAD51C/D, BRCA1/2	8 /12

D)

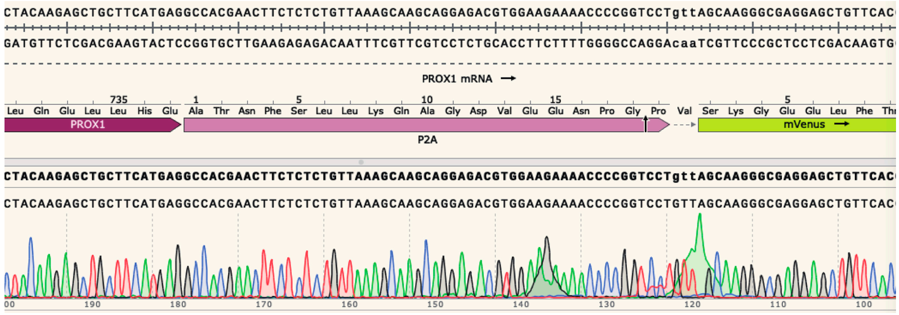

Figure S5. Gene targeting in human PROX1 locus. (**A**) Graphical schematic of PROX1-Venus KI. Gray arrows indicate primer biding sites for genotyping. (**B**) Representative results of PROX1-Venus KI using Cas9/sgRNA with or without the overexpression vectors of *RAD51C/D* and *BRCA1/2*. (**C**) Summary of KI experiment results using the PROX1-Venus targeting vector with or without the HR factor-overexpressing vectors. (**D**) DNA sequencing analysis of the PROX-Venus KI allele in the vicinity of the last codon.

Figure S6

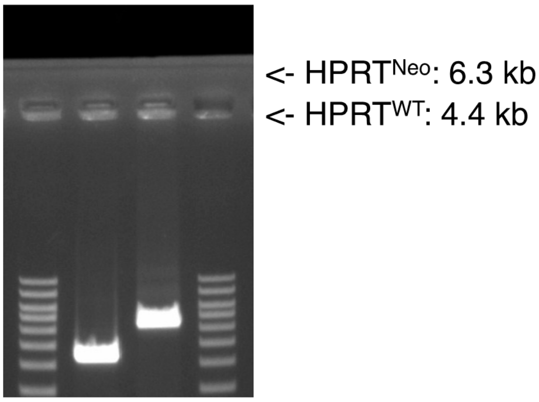

Figure S6. Genotyping PCR analysis of WT (left lane) and G418/6-TG-resistant iPSCs (right lane) in the *HPRT* locus. PCR was performed using two specific primers (CTGGCTGGGTCCTAGTTTATGCT and ATGCATAGCCAGTGCTTGAGAAG). Primer binding sites are also shown in Supplementary Sequence.

Figure S7

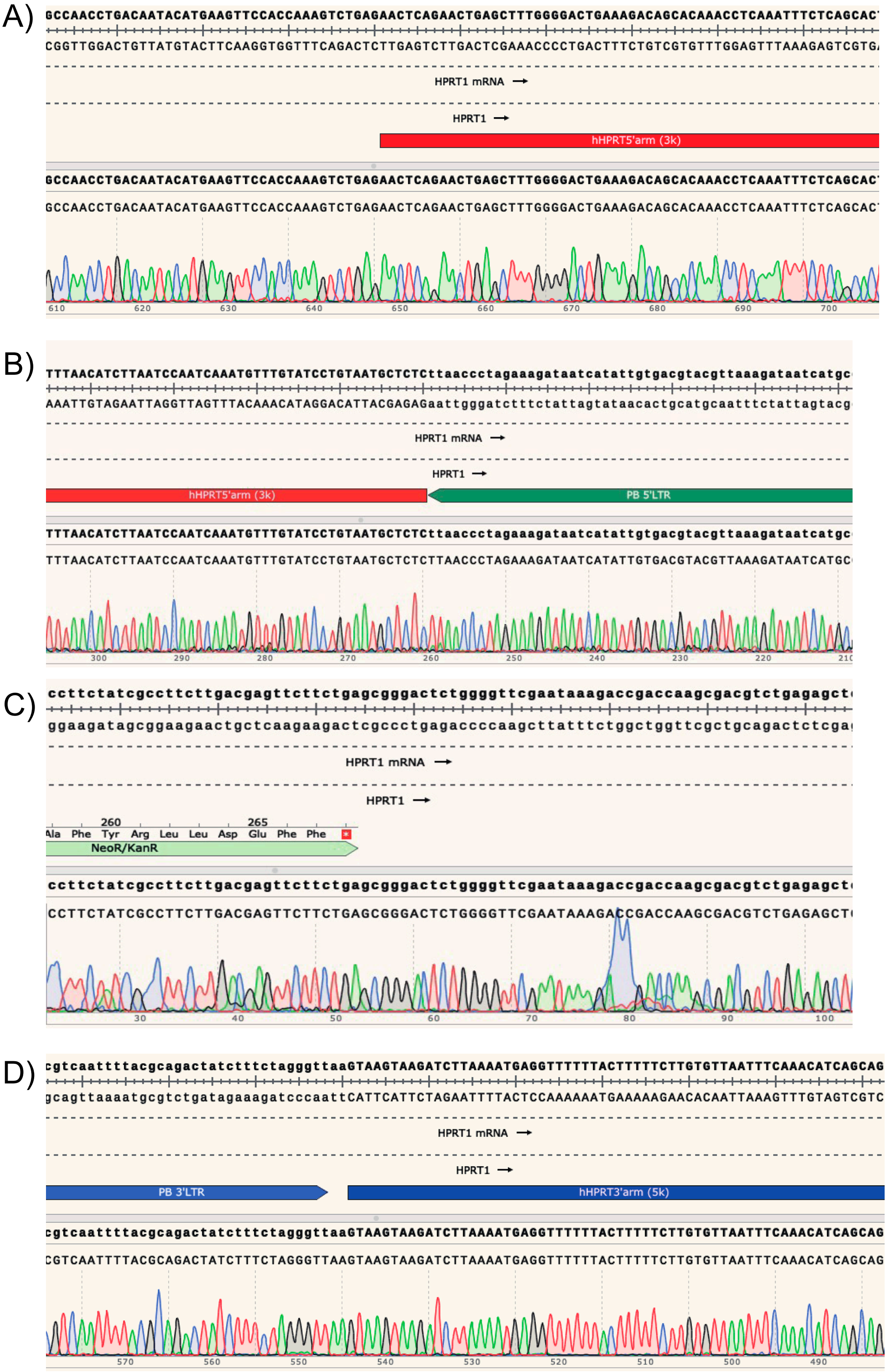

Figure S7. DNA sequencing analysis of the PCR-amplified HPRT^{Neo} allele in G418 and 6-

TG resistant iPSCs (shown in Figure S7). (**A**) Sequencing of the vicinity of the junction of the 5' homology arm and external region. (**B**) Sequencing of the vicinity of the direct upstream of the *piggyBac* 5'LTR (the *PGK-Neo* cassette shown in Figure 3A is flanked by *piggyBac* LTRs). (**C**) Sequencing of the vicinity of the termination codon of the *Neomycin-resistance* gene. (**D**) Sequencing of the vicinity of the direct downstream of the *piggyBac* 3'LTR. Full sequencing alignment data are available in Supplementary Sequence.

Figure S8

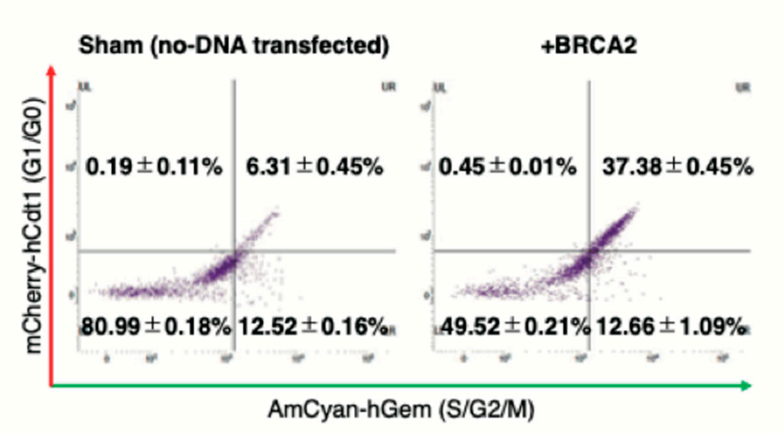

Figure S8. Cell cycle analysis using FACS and Fucci2.1-transfected etKA iPSCs. The iPSCs were transfected with the BRCA2 overexpression vector (+BRCA2) or without (sham; only TE buffer was used for transfection).

Supplementary Discussion

RAD51 and *DMC1* are the eukaryotic homologues of bacterial *RecA* and archaeal *RadA* [2,3] and are well-known key factors for the HR pathway [4]. The RAD51 protein forms a nucleoprotein filament on single-stranded DNA (ssDNA) to promote DNA-homology search, recognition, and strand exchange [5] by exploiting two motifs critical for homology search and subsequent recombination [6]. The overexpression of *RAD51* reportedly contributes to the enhancement of the HR ratio in multiple cell lines (reviewed in [7]), but there is considerable variation in the extent of this effect depending on experimental settings and cell lines. [8], reported that *RAD51* overexpression in hPSCs did not result in a significant enhancement of the HR ratio in CRISPR-Cas9 mediated gene editing, but did cause an increase in biallelic KI. The latter might have resulted from the putative enhancement of RAD51 overexpression-induced inter-homolog repair [9], classically known as sister-chromatid exchange [10,11]. Our results on *RAD51* overexpression here were not fully consistent with the data from Takayama et al.

(2017). This disparity might have resulted from the presence of different rate-limiting factors for HDR/NHEJ choice in different loci, cell types and lines.

From prokaryotes to eukaryotes, additional *RecA*-like genes have evolved following multiple duplication events and divergent evolution of the ancestral gene [3,12]. In vertebrate species, five genes have been identified as *RecA*/*RAD51* paralogues, namely, *RAD51B*, *RAD51C*, and *RAD51D* by homology searches [13-15], and *XRCC2* and *XRCC3* by their ability to complement susceptibility to ionizing radiation in the Chinese hamster CHO cell line [16,17]. Structural studies revealed that the *RecA/RAD51* paralogues function mainly through formation of two distinct complexes (i) the RAD51C-XRCC3 complex, and (ii) the RAD51B-RAD51C-RAD51D-XRCC2 complex [18,19]. Respective knock-outs of these *RecA*/*RAD51* homologues in cell lines result in defective cell growth and HR, and hypersensitivity to DNA-damaging agents [20]. Moreover, mice with mutations of the *RecA*/*RAD51* homologues show embryonic lethality [21,22], indicating their importance for DNA repair, genomic stability, and cellular viability. On the other hand, the effect of overexpressing these paralogues has not been thoroughly investigated and nor has their expression patterns *in vivo*. In this context, our results showed, for the first time, that overexpressing these paralogues could enhance the efficiency of targeted gene engineering.

Heterozygous *BRCA1/2* mutations confer a high risk for breast and ovarian cancer in women [23-28]. Moreover, cell lines and mouse mutants for these genes show various deficiencies, including hypersensitivity to irradiation, increased tumorigenesis, and HR deficiency; mouse embryos homozygous for *Brca1* and *Brca2* null mutations (*Brca1*$^{-/-}$ and *Brca2*$^{-/-}$) show developmental arrest during fetal development [29,30]. As tumorigenesis in patients carrying inherited heterozygous *BRCA1/2* mutations is triggered by deactivation of the WT allele, *BRCA1/2* are generally considered as tumor-suppressor genes.

The BRCA1 protein binds to many cellular proteins *in vivo*. For example, the BRCT domain in the C-terminus of BRCA1 recognizes a variety of DNA repair proteins [31]. The BRCA1 protein colocalizes with RAD51 in nuclear foci in mitotic cells [32]; this colocalization is essential for HR upon replication fork stalling and collapse [33]. Additionally, via interaction with CtBP-interacting protein (CtIP), BRCA1 promotes ssDNA resection that is critical for NHEJ/HDR choice by competing with the HDR/MMEJ inhibitor protein P53BP1 [34]. Recent studies showed that knock-in (HDR) efficiencies with CRISPR-Cas9 were enhanced by direct or indirect suppression of P53BP1 activity [35,36]. Thus, the P53BP1-related mechanism provides a possible explanation for the ability of BRCA1 overexpression to enhance the HR ratio in hiPSCs.

The BRCA2 protein interacts directly with RAD51 through the BRC domain [37] and the C-terminus [38]. BRCA2 is required for recruitment of RAD51 to DSB sites, and is also important for its tumor-suppressive function [39]. BRCA2 also has a DNA-binding domain that binds both ssDNA and dsDNA. These domains are thought to facilitate the formation of RAD51 nucleofilaments in HR. As a secondary role, BRCA2 may protect nascent DNA by MRE11A-mediated degradation upon replication fork stalling [40]. These various properties clearly stress the importance of BRCA2 for HR and genomic stability; however, few studies have examined the effects of overexpression of *BRCA2* on HR. [41] reported that *BRCA2* overexpression, especially its cancer-associated variants, enhances spontaneous HR in HeLa cells; whether this effect occurs in other cell lines has not been thoroughly investigated. In this context, this study provides the first evidence that *BRCA2* overexpression in hPSCs has an HR-stimulating effect; this property might contribute to the development of stem cell-based technologies and therapies.

Supplementary References

1. Yoshimatsu, S.; Sone, T.; Nakajima, M.; Sato, T.; Okochi, R.; Ishikawa, M.; Nakamura, M.; Sasaki, E.; Shiozawa, S.; Okano, H. A versatile toolbox for knock-in gene targeting based on the Multisite Gateway technology. *PLoS One* **2019**, *14*, e0221164, doi:10.1371/journal.pone.0221164.
2. Chen, Z.; Yang, H.; Pavletich, N.P. Mechanism of homologous recombination from the RecA-ssDNA/dsDNA structures. *Nature* **2008**, *453*, 489-484, doi:10.1038/nature06971.
3. Lin, Z.; Kong, H.; Nei, M.; Ma, H. Origins and evolution of the recA/RAD51 gene family: evidence for ancient gene duplication and endosymbiotic gene transfer. *Proc Natl Acad Sci U S A* **2006**, *103*, 10328-10333, doi:10.1073/pnas.0604232103.
4. Baumann, P.; West, S.C. Role of the human RAD51 protein in homologous recombination and double-stranded-break repair. *Trends Biochem Sci* **1998**, *23*, 247-251, doi:10.1016/s0968-0004(98)01232-8.
5. Bianco, P.R.; Tracy, R.B.; Kowalczykowski, S.C. DNA strand exchange proteins: a biochemical and physical comparison. *Front Biosci* **1998**, *3*, D570-603, doi:10.2741/a304.
6. Ito, K.; Murayama, Y.; Kurokawa, Y.; Kanamaru, S.; Kokabu, Y.; Maki, T.; Mikawa, T.; Argunhan, B.; Tsubouchi, H.; Ikeguchi, M., et al. Real-time tracking reveals catalytic roles for the two DNA binding sites of Rad51. *Nat Commun* **2020**, *11*, 2950, doi:10.1038/s41467-020-16750-3.
7. Klein, H.L. The consequences of Rad51 overexpression for normal and tumor cells. *DNA Repair (Amst)* **2008**, *7*, 686-693, doi:10.1016/j.dnarep.2007.12.008.
8. Takayama, K.; Igai, K.; Hagihara, Y.; Hashimoto, R.; Hanawa, M.; Sakuma, T.; Tachibana, M.; Sakurai, F.; Yamamoto, T.; Mizuguchi, H. Highly efficient biallelic genome editing

of human ES/iPS cells using a CRISPR/Cas9 or TALEN system. *Nucleic Acids Res* **2017**, *45*, 5198-5207, doi:10.1093/nar/gkx130.
9. Wilde, J.J.; Aida, T.; Wienisch, M.; Zhang, Q.; Qi, P.; Feng, G. Efficient Zygotic Genome Editing via RAD51-Enhanced Interhomolog Repair. *bioRxiv* **2018**, 10.1101/263699, 263699, doi:10.1101/263699.
10. Painter, R.B. A replication model for sister-chromatid exchange. *Mutat Res* **1980**, *70*, 337-341, doi:10.1016/0027-5107(80)90023-8.
11. Sonoda, E.; Sasaki, M.S.; Morrison, C.; Yamaguchi-Iwai, Y.; Takata, M.; Takeda, S. Sister chromatid exchanges are mediated by homologous recombination in vertebrate cells. *Mol Cell Biol* **1999**, *19*, 5166-5169, doi:10.1128/mcb.19.7.5166.
12. Thacker, J. The role of homologous recombination processes in the repair of severe forms of DNA damage in mammalian cells. *Biochimie* **1999**, *81*, 77-85, doi:10.1016/s0300-9084(99)80041-8.
13. Pittman, D.L.; Weinberg, L.R.; Schimenti, J.C. Identification, characterization, and genetic mapping of Rad51d, a new mouse and human RAD51/RecA-related gene. *Genomics* **1998**, *49*, 103-111, doi:10.1006/geno.1998.5226.
14. Albala, J.S.; Thelen, M.P.; Prange, C.; Fan, W.; Christensen, M.; Thompson, L.H.; Lennon, G.G. Identification of a novel human RAD51 homolog, RAD51B. *Genomics* **1997**, *46*, 476-479, doi:10.1006/geno.1997.5062.
15. Dosanjh, M.K.; Collins, D.W.; Fan, W.; Lennon, G.G.; Albala, J.S.; Shen, Z.; Schild, D. Isolation and characterization of RAD51C, a new human member of the RAD51 family of related genes. *Nucleic Acids Res* **1998**, *26*, 1179-1184, doi:10.1093/nar/26.5.1179.
16. Tebbs, R.S.; Zhao, Y.; Tucker, J.D.; Scheerer, J.B.; Siciliano, M.J.; Hwang, M.; Liu, N.; Legerski, R.J.; Thompson, L.H. Correction of chromosomal instability and sensitivity to diverse mutagens by a cloned cDNA of the XRCC3 DNA repair gene. *Proc Natl Acad Sci U S A* **1995**, *92*, 6354-6358, doi:10.1073/pnas.92.14.6354.
17. Cartwright, R.; Tambini, C.E.; Simpson, P.J.; Thacker, J. The XRCC2 DNA repair gene from human and mouse encodes a novel member of the recA/RAD51 family. *Nucleic Acids Res* **1998**, *26*, 3084-3089, doi:10.1093/nar/26.13.3084.
18. Liu, N.; Schild, D.; Thelen, M.P.; Thompson, L.H. Involvement of Rad51C in two distinct protein complexes of Rad51 paralogs in human cells. *Nucleic Acids Res* **2002**, *30*, 1009-1015, doi:10.1093/nar/30.4.1009.
19. Braybrooke, J.P.; Spink, K.G.; Thacker, J.; Hickson, I.D. The RAD51 family member, RAD51L3, is a DNA-stimulated ATPase that forms a complex with XRCC2. *J Biol Chem* **2000**, *275*, 29100-29106, doi:10.1074/jbc.M002075200.
20. Takata, M.; Sasaki, M.S.; Tachiiri, S.; Fukushima, T.; Sonoda, E.; Schild, D.; Thompson,

L.H.; Takeda, S. Chromosome instability and defective recombinational repair in knockout mutants of the five Rad51 paralogs. *Mol Cell Biol* **2001**, *21*, 2858-2866, doi:10.1128/MCB.21.8.2858-2866.2001.

21. Thompson, L.H.; Schild, D. Homologous recombinational repair of DNA ensures mammalian chromosome stability. *Mutat Res* **2001**, *477*, 131-153, doi:10.1016/s0027-5107(01)00115-4.
22. Kuznetsov, S.G.; Haines, D.C.; Martin, B.K.; Sharan, S.K. Loss of Rad51c leads to embryonic lethality and modulation of Trp53-dependent tumorigenesis in mice. *Cancer Res* **2009**, *69*, 863-872, doi:10.1158/0008-5472.CAN-08-3057.
23. Miki, Y.; Swensen, J.; Shattuck-Eidens, D.; Futreal, P.A.; Harshman, K.; Tavtigian, S.; Liu, Q.; Cochran, C.; Bennett, L.M.; Ding, W., et al. A strong candidate for the breast and ovarian cancer susceptibility gene BRCA1. *Science* **1994**, *266*, 66-71, doi:10.1126/science.7545954.
24. Lancaster, J.M.; Wooster, R.; Mangion, J.; Phelan, C.M.; Cochran, C.; Gumbs, C.; Seal, S.; Barfoot, R.; Collins, N.; Bignell, G., et al. BRCA2 mutations in primary breast and ovarian cancers. *Nat Genet* **1996**, *13*, 238-240, doi:10.1038/ng0696-238.
25. Futreal, P.A.; Liu, Q.; Shattuck-Eidens, D.; Cochran, C.; Harshman, K.; Tavtigian, S.; Bennett, L.M.; Haugen-Strano, A.; Swensen, J.; Miki, Y., et al. BRCA1 mutations in primary breast and ovarian carcinomas. *Science* **1994**, *266*, 120-122, doi:10.1126/science.7939630.
26. Wooster, R.; Bignell, G.; Lancaster, J.; Swift, S.; Seal, S.; Mangion, J.; Collins, N.; Gregory, S.; Gumbs, C.; Micklem, G. Identification of the breast cancer susceptibility gene BRCA2. *Nature* **1995**, *378*, 789-792, doi:10.1038/378789a0.
27. Ford, D.; Easton, D.F.; Bishop, D.T.; Narod, S.A.; Goldgar, D.E. Risks of cancer in BRCA1-mutation carriers. Breast Cancer Linkage Consortium. *Lancet* **1994**, *343*, 692-695, doi:10.1016/s0140-6736(94)91578-4.
28. Peto, J.; Collins, N.; Barfoot, R.; Seal, S.; Warren, W.; Rahman, N.; Easton, D.F.; Evans, C.; Deacon, J.; Stratton, M.R. Prevalence of BRCA1 and BRCA2 gene mutations in patients with early-onset breast cancer. *J Natl Cancer Inst* **1999**, *91*, 943-949, doi:10.1093/jnci/91.11.943.
29. Shen, S.X.; Weaver, Z.; Xu, X.; Li, C.; Weinstein, M.; Chen, L.; Guan, X.Y.; Ried, T.; Deng, C.X. A targeted disruption of the murine Brca1 gene causes gamma-irradiation hypersensitivity and genetic instability. *Oncogene* **1998**, *17*, 3115-3124, doi:10.1038/sj.onc.1202243.
30. Sharan, S.K.; Morimatsu, M.; Albrecht, U.; Lim, D.S.; Regel, E.; Dinh, C.; Sands, A.; Eichele, G.; Hasty, P.; Bradley, A. Embryonic lethality and radiation hypersensitivity

mediated by Rad51 in mice lacking Brca2. *Nature* **1997**, *386*, 804-810, doi:10.1038/386804a0.
31. Deng, C.X.; Brodie, S.G. Roles of BRCA1 and its interacting proteins. *Bioessays* **2000**, *22*, 728-737, doi:10.1002/1521-1878(200008)22:8<728::AID-BIES6>3.0.CO;2-B.
32. Scully, R.; Chen, J.; Plug, A.; Xiao, Y.; Weaver, D.; Feunteun, J.; Ashley, T.; Livingston, D.M. Association of BRCA1 with Rad51 in mitotic and meiotic cells. *Cell* **1997**, *88*, 265-275, doi:10.1016/s0092-8674(00)81847-4.
33. Feng, Z.; Zhang, J. A dual role of BRCA1 in two distinct homologous recombination mediated repair in response to replication arrest. *Nucleic Acids Res* **2012**, *40*, 726-738, doi:10.1093/nar/gkr748.
34. Chapman, J.R.; Sossick, A.J.; Boulton, S.J.; Jackson, S.P. BRCA1-associated exclusion of 53BP1 from DNA damage sites underlies temporal control of DNA repair. *J Cell Sci* **2012**, *125*, 3529-3534, doi:10.1242/jcs.105353.
35. Jayavaradhan, R.; Pillis, D.M.; Goodman, M.; Zhang, F.; Zhang, Y.; Andreassen, P.R.; Malik, P. CRISPR-Cas9 fusion to dominant-negative 53BP1 enhances HDR and inhibits NHEJ specifically at Cas9 target sites. *Nat Commun* **2019**, *10*, 2866, doi:10.1038/s41467-019-10735-7.
36. Nambiar, T.S.; Billon, P.; Diedenhofen, G.; Hayward, S.B.; Taglialatela, A.; Cai, K.; Huang, J.W.; Leuzzi, G.; Cuella-Martin, R.; Palacios, A., et al. Stimulation of CRISPR-mediated homology-directed repair by an engineered RAD18 variant. *Nat Commun* **2019**, *10*, 3395, doi:10.1038/s41467-019-11105-z.
37. Yang, H.; Li, Q.; Fan, J.; Holloman, W.K.; Pavletich, N.P. The BRCA2 homologue Brh2 nucleates RAD51 filament formation at a dsDNA-ssDNA junction. *Nature* **2005**, *433*, 653-657, doi:10.1038/nature03234.
38. Esashi, F.; Christ, N.; Gannon, J.; Liu, Y.; Hunt, T.; Jasin, M.; West, S.C. CDK-dependent phosphorylation of BRCA2 as a regulatory mechanism for recombinational repair. *Nature* **2005**, *434*, 598-604, doi:10.1038/nature03404.
39. Moynahan, M.E.; Pierce, A.J.; Jasin, M. BRCA2 is required for homology-directed repair of chromosomal breaks. *Mol Cell* **2001**, *7*, 263-272, doi:10.1016/s1097-2765(01)00174-5.
40. Schlacher, K.; Christ, N.; Siaud, N.; Egashira, A.; Wu, H.; Jasin, M. Double-strand break repair-independent role for BRCA2 in blocking stalled replication fork degradation by MRE11. *Cell* **2011**, *145*, 529-542, doi:10.1016/j.cell.2011.03.041.
41. Balia, C.; Galli, A.; Caligo, M.A. Effect of the overexpression of BRCA2 unclassified missense variants on spontaneous homologous recombination in human cells. *Breast Cancer Res Treat* **2011**, *129*, 1001-1009, doi:10.1007/s10549-011-1607-y.
